# Supplementary material for: The causal relationship between the human gut microbiota and pyogenic arthritis: a Mendelian randomization study
Source: Front Cell Infect Microbiol. 2024 Nov 26;14:1452480. doi: 10.3389/fcimb.2024.1452480 (PMC11629706; doi:10.3389/fcimb.2024.1452480)
Supplement: Supplementary file 1 [file DataSheet1.zip › mendelian test/ebi-a-GCST90027776.csv_forest.pdf]

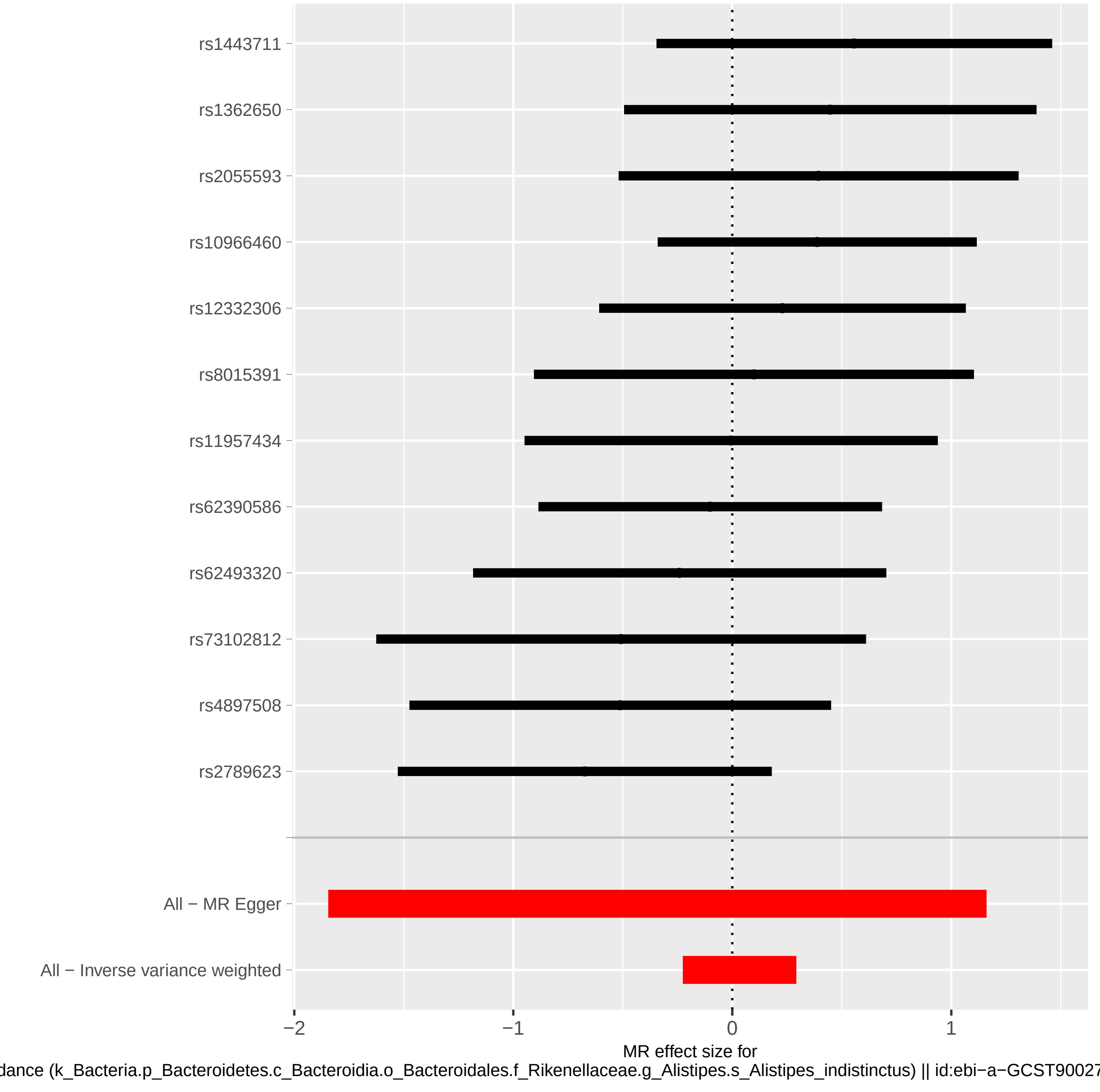
rs1443711
rs1362650
rs2055593
rs10966460
rs12332306
rs8015391
rs11957434
rs62390586
rs62493320
rs73102812
rs4897508
rs2789623
All − MR Egger
All − Inverse variance weighted
−2
−1
0
1
MR effect size for
dance (k_Bacteria.p_Bacteroidetes.c_Bacteroidia.o_Bacteroidales.f_Rikenellaceae.g_Alistipes.s_Alistipes_indistinctus) || id:ebi−a−GCST90027
